# O.1.1-8 Impact on health-related quality of life after a concurrent exercise program in adults with treatment resistant depression: TRACE-RMD study

**DOI:** 10.1093/eurpub/ckad133.084

**Published:** 2023-09-11

**Authors:** José Etxaniz-Osés, Mikel Tous-Espelosin, Nagore Iriarte-Yoller, Sara Maldonado-Martin

**Affiliations:** Department of Physical Education and Sport, Faculty of Education and Sport-Physical Activity and Sport Sciences Section, University of the Basque Country (UPV/EHU), Vitoria-Gasteiz, Araba/Álava, Basque Country, Spain; Gizartea, Kirola eta Ariketa Fisikoa Ikerkuntza Taldea (GIKAFIT), Society, Sports, and Physical Exercise Research Group, Department of Physical Education and Sport, Faculty of Education and Sport- Physical Activity and Sport Sciences Section, University of the Basque Country (UPV/EHU), Vitoria-Gasteiz, Araba/Álava, Basque Country, Spain; Bioaraba Health Research Institute, Physical Activity, Exercise, and Health Group, Vitoria-Gasteiz. Araba/Álava, Basque Country, Spain; Bioaraba Health Research Institute, New Therapies in Mental Health Group, Vitoria-Gasteiz, Spain, Osakidetza Basque Health Service, Araba Mental Health Network, Psychiatric Hospital of Alava, Vitoria-Gasteiz, Spain; Department of Medicine, Faculty of Health Sciences, University of Deusto, Bilbao, Spain; jechaniz012@ikasle.ehu.eus; Gizartea, Kirola eta Ariketa Fisikoa Ikerkuntza Taldea (GIKAFIT), Society, Sports, and Physical Exercise Research Group, Department of Physical Education and Sport, Faculty of Education and Sport- Physical Activity and Sport Sciences Section, University of the Basque Country (UPV/EHU), Vitoria-Gasteiz, Araba/Álava, Basque Country, Spain; Bioaraba Health Research Institute, Physical Activity, Exercise, and Health Group, Vitoria-Gasteiz. Araba/Álava, Basque Country, Spain

## Abstract

**Purpose:**

Treatment-resistant depression (TRD) is defined as those who do not remit to major depressive disorder with pharmacological treatment. TRD is associated with a worse health-related quality of life (QoL). Thus, people with TRD have reported greater disability than those healthy control (HC) in QoL, assessed by the 36-Item Short-Form Health Survey questionnaire (SF-36). Concurrent exercise training (i.e., a combination of aerobic and resistance exercise in the same session) could be an efficient non-pharmacological treatment strategy for improving QoL. Exercise is recommended as a non-pharmacological adjuvant program for patients with TRD. Therefore, this study aimed to determine the effectiveness of a concurrent exercise program on QoL in a TRD population.

**Methods:**

Participants (n = 15, 73.3% women, 57.7±13.7 yr old) with TRD carried out a concurrent exercise program (2 days/week and 12 weeks of intervention). The SF-36 questionnaire was used to assess health-related QoL. At baseline, the TRD population showed lower scores (P ≤ 0.001) in physical function, role-physical, bodily pain, general health, vitality, social functioning, emotional role, mental health, and physical and mental component summaries compared to HC.

**Results:**

After 12-week of intervention, general health (↑50.5%), vitality (↑41.2%), social functioning (↑76.4%), and mental component summary (↑24.9%) were increased (P < 0.05). However, there was not a significant (P > 0.05) change increase in physical functioning, role physical, bodily pain, emotional role, mental health, and physical component summary.

**Conclusion:**

In conclusion, beneficial effects on QoL variables in patients with TRD after concurrent exercise should lead to considering exercise as an adjuvant program in their treatment.

